# Persistent symptom burden in long-standing systemic lupus erythematosus: a subgroup-focused hypothesis for an SLE–ME/CFS research phenotype

**DOI:** 10.1186/s12967-026-08867-8

**Published:** 2026-09-25

**Authors:** Lotte Habermann-Horstmeier

**Affiliations:** Villingen Institute of Public Health (VIPH), Klosterring 5, D-78050 Villingen-Schwenningen, Germany

## Abstract

**Background:**

Despite improved control of inflammatory disease activity in systemic lupus erythematosus (SLE), many patients experience persistent fatigue, impaired physical function, cognitive symptoms, sleep disturbance, and reduced exercise tolerance, including during remission. These symptoms are multifactorial and may not be adequately explained by conventional measures of inflammatory activity. Clinical and biological parallels with myalgic encephalomyelitis/chronic fatigue syndrome (ME/CFS) suggest that interacting immune, metabolic, vascular, and autonomic mechanisms may contribute to persistent symptom burden in a subgroup of patients with long-standing SLE.

**Objective:**

To develop a subgroup-focused, non-linear, and testable framework for persistent symptoms in SLE by integrating established SLE evidence with carefully distinguished mechanistic insights from ME/CFS research.

**Methods:**

This Commentary is based on a targeted, non-systematic literature synthesis of publications identified in PubMed and Web of Science. Evidence was categorized as direct SLE evidence, evidence from ME/CFS, or cross-condition mechanistic inference. Priority was given to human studies, systematic reviews, meta-analyses, and translational research addressing persistent symptoms, autonomic and endothelial dysfunction, mitochondrial and redox alterations, and neurocognitive manifestations.

**Results:**

We propose a hypothesized SLE–ME/CFS research phenotype characterized by persistent, functionally limiting symptoms despite low inflammatory disease activity or remission. The phenotype includes fatigue lasting at least six months together with post-exertional symptom exacerbation, unrefreshing sleep, cognitive symptoms, and/or orthostatic intolerance, following structured exclusion or assessment of alternative explanations and comorbidities. Direct SLE evidence supports the relevance of residual immune activation, oxidative stress, mitochondrial alterations, endothelial dysfunction, and dysautonomia. Findings from ME/CFS further suggest potentially convergent mechanisms involving impaired cellular energetics, autonomic dysfunction, altered cerebral perfusion, and neuroimmune dysregulation. However, these cross-condition inferences require direct validation in SLE.

**Conclusions:**

Persistent symptom burden in long-standing SLE may reflect a biologically distinct subgroup in which immune, autonomic, vascular, metabolic, and neurocognitive mechanisms interact dynamically. The proposed framework does not define a new diagnostic entity or fixed disease trajectory but provides a hypothesis-generating basis for prospective longitudinal studies. Multidimensional phenotyping may improve patient stratification, biomarker discovery, and supportive care for patients whose symptoms remain disproportionate to conventional measures of disease activity.

## Introduction

Systemic lupus erythematosus (SLE) is a clinically and biologically heterogeneous autoimmune disease in which autoantibody production, immune-complex formation, complement activation, and type I interferon-related immune dysregulation contribute to systemic inflammation and organ involvement [1–3]. Major therapeutic advances have improved survival and the control of inflammatory disease activity. Nevertheless, a substantial proportion of patients continue to experience persistent fatigue, impaired physical functioning, cognitive symptoms, sleep disturbance, and reduced exercise tolerance, including during periods of low conventional disease activity or clinical remission [4–6]. This discordance between inflammatory disease control and patient-reported symptom burden represents an important unresolved clinical problem in SLE.

Persistent symptoms in SLE are usually multifactorial. They may reflect active or residual immune processes, accumulated organ damage, pain, sleep disturbance, anaemia, endocrine dysfunction, medication effects, depression, fibromyalgia, or deconditioning. At the same time, the persistence of substantial functional impairment in some patients despite apparently controlled inflammatory activity raises the possibility that additional, interacting regulatory mechanisms contribute to symptom maintenance. In particular, immune dysregulation, oxidative and metabolic stress, endothelial and microvascular abnormalities, and autonomic dysfunction may represent interconnected domains that are insufficiently captured by conventional inflammatory disease activity indices [7–10].

This question is especially relevant in view of conceptual and biological parallels with myalgic encephalomyelitis/chronic fatigue syndrome (ME/CFS). ME/CFS is characterised by severe functional impairment, post-exertional malaise, unrefreshing sleep, cognitive dysfunction, and orthostatic intolerance, and research increasingly points towards interacting abnormalities in immune signalling, cellular energetics, autonomic regulation, vascular function, and cerebral perfusion [11–15]. Several of these domains are also biologically plausible in SLE. Their coexistence may be particularly relevant in patients with long-standing disease and persistent symptom burden that is disproportionate to conventional inflammatory measures.

We therefore propose that a subgroup of patients with established SLE may exhibit a distinct persistent-symptom phenotype with ME/CFS-relevant clinical and biological features. This proposed phenotype is not defined by fatigue alone. It is characterised by persistent functional limitation despite low inflammatory disease activity or remission, together with a pattern of exertional symptom exacerbation, cognitive symptoms, unrefreshing sleep, orthostatic intolerance, or a combination of these manifestations [10, 16]. Sleep disturbance is common in SLE and is associated with fatigue and broader physical and psychological burden [17, 18]. We hypothesise that, in susceptible individuals, residual immune dysregulation, metabolic and redox stress, vascular or endothelial dysfunction, and autonomic disturbance may interact bidirectionally and contribute to a self-perpetuating symptom burden [7–10, 19]. Potential susceptibility factors may include genetic predisposition and epigenetic background, cumulative inflammatory burden, vascular vulnerability, autonomic phenotype, or environmental induced physical and psychological modifiers, but these remain to be established. The relative contribution of these mechanisms is likely to vary between individuals and may change over the course of disease.

SLE is a particularly informative model for investigating this hypothesis. Its immune-complex biology, type I interferon signature, vascular involvement, and neuropsychiatric manifestations provide disease-specific contexts in which links between immune activity, systemic regulation, and functional impairment can be studied. Importantly, SLE also permits the investigation of persistent symptoms against the background of established clinical measures of inflammatory activity, organ involvement, and immunological biomarkers. This may help distinguish symptom phenotypes related predominantly to active inflammation or organ damage from those associated with broader dysregulation across immune, autonomic, vascular, and metabolic systems. Whether comparable phenotypes occur in other autoimmune diseases, such as rheumatoid arthritis or primary Sjögren’s syndrome, is an important question for comparative research rather than a reason to dismiss the potential value of SLE as a model disease.

## Objective

The aim of this Commentary is to develop a subgroup-focused, non-linear, and testable framework for persistent symptom burden in long-standing SLE. We integrate established findings from SLE with evidence from ME/CFS research to identify convergent mechanisms, clearly distinguish direct evidence from cross-condition inference, and propose priorities for prospective phenotyping. These include structured assessment of alternative explanations and comorbidities, evaluation of post-exertional symptom exacerbation, autonomic and vascular testing, and exploratory immune and metabolic biomarker studies. This subgroup is defined by convergent clinical and biological characteristics rather than by SLE diagnosis alone. The proposed framework is intended to stimulate longitudinal research on clinically meaningful subgroups, susceptibility factors, and biological markers that may ultimately improve patient stratification and supportive care.

## Methods

This Commentary is based on a targeted, non-systematic literature synthesis. Relevant publications were identified through structured searches of PubMed and Web of Science between February 15 and July 29, 2026, using combinations of search terms related to SLE, persistent fatigue, autonomic dysfunction, endothelial dysfunction, mitochondrial dysfunction, neurocognitive symptoms, and ME/CFS (Table 1). Priority was given to human studies, systematic reviews, meta-analyses, recent narrative reviews, and translational primary studies addressing pathophysiological mechanisms, particularly those including patients with low disease activity or clinical remission. Additional considerations included diagnostic criteria, clinical population, multisystem relevance, and methodological quality. Publications addressing non-specific fatigue (e.g., cancer-related fatigue) and studies relying exclusively on outdated ME/CFS case definitions (e.g., Fukuda criteria) were not considered.

**Table 1:** Search terms used in the literature search (Representative boolean combinations included)

Focus	Example search terms
Disease entities	“Systemic Lupus Erythematosus” OR “SLE”; “ME/CFS” OR “Myalgic Encephalomyelitis” OR “Chronic Fatigue Syndrome”
Persistent symptoms/Clinical manifestations	“persistent fatigue”; “chronic fatigue”; “post-exertional malaise”; “exercise intolerance”; “orthostatic intolerance”; “brain fog”; “cognitive impairment”; “sleep disturbance”;
Biological mechanisms	“autonomic dysfunction”; “dysautonomia”; “POTS”; “endothelial dysfunction”; “microvascular”; “mitochondrial”; “bioenergetics”; “oxidative stress”; “redox”; “type I interferon”; “immunometabolism”
Cross-condition searches	(“SLE” AND “autonomic dysfunction”); (“SLE” AND mitochondrial); (“SLE” AND “endothelial dysfunction”); (“SLE” AND “fatigue”); (“SLE” AND “remission” AND “fatigue”); (“SLE AND autoantibodies”); (“ME/CFS” AND “Systemic Lupus Erythematosus”); (“ME/CFS” AND “endothelial dysfunction”); “ME/CFS” AND “cerebral blood flow”); (“ME/CFS” AND autoantibodies”); (“ME/CFS AND autonomic dysfunction”)

The evidence was synthesised according to three categories: (i) direct evidence from SLE, (ii) evidence from ME/CFS, and (iii) cross-condition mechanistic inference. Throughout the manuscript, direct SLE evidence is presented separately from extrapolation based on ME/CFS, and mechanistic inference is explicitly identified where appropriate. Because the objective was conceptual synthesis rather than systematic evidence appraisal, no formal quality assessment or risk-of-bias evaluation was performed. Where findings differed between studies, potential sources of heterogeneity, including disease duration, disease activity, sex distribution, and study population, were considered during interpretation.

Additional considerations during study selection included multisystem relevance, diagnostic criteria, study population, disease stage, and study design.

## Results

### Clinical overlap between SLE and ME/CFS and unmet clinical need

Fatigue, cognitive symptoms, sleep disturbances, and orthostatic symptoms do not, on their own, confirm the presence of an ME/CFS phenotype. Taken together, however, they provide an initial indication of this. They must be distinguished from active SLE, organ damage, pain syndromes, sleep disturbances, depression, fibromyalgia, anemia, endocrine causes, medication effects, and deconditioning, if there are further indications that these conditions are present. These symptoms require structured assessment to distinguish them from active disease, organ damage, anaemia, endocrine disorders, medication effects, fibromyalgia, depression, sleep disorders, or deconditioning. Such assessment is essential before considering a persistent symptom phenotype with ME/CFS-relevant characteristics.

Taken together, these observations indicate that a subgroup of SLE patients exhibits a symptom profile extending beyond classical inflammatory manifestations and overlapping with features of systemic dysregulation. This supports the hypothesis that additional pathophysiological mechanisms contribute to long-term disease burden.

### A subgroup-focused, non-linear pathophysiological model

The persistence of disabling symptoms in SLE despite apparently controlled inflammatory activity suggests that underlying mechanisms extend beyond immune-mediated inflammation. Instead, a dynamic interplay between immune activation, metabolic regulation, vascular function, and autonomic control may evolve over time. These mechanisms can occur simultaneously, reinforcing or counteracting one another and in which their relative importance varies from person to person and depending on the stage of the disease.

We therefore propose a subgroup-focused, non-linear conceptual model in which persistent symptoms in a subset of patients with long-standing SLE arise from interacting immune, autonomic, vascular, metabolic, and neurocognitive mechanisms whose relative contributions vary between individuals and over time.

#### Established SLE-related mechanisms and symptom correlates

In early SLE, disease activity is driven by well-characterized immunological mechanisms, including autoantibody production, immune complex formation, complement activation, and a type I interferon response [2, 3, 20]. These processes result in systemic inflammation and organ involvement. Even during clinical remission, residual immune activation and low-grade inflammation may persist.

With ongoing disease, repeated or persistent immune activation may contribute to cumulative biological stress and the gradual emergence of non-inflammatory mechanisms of dysfunction. In this context, chronic inflammatory signaling can induce sustained metabolic reprogramming that alters mitochondrial function, redox balance, and substrate utilization, thereby suggesting a close link between immune activity and systemic energy availability [21, 22]. This is consistent with evidence of increased oxidative stress and redox dysregulation in SLE T cells [7]. Evidence from SLE and related conditions further demonstrates microvascular dysfunction and impaired endothelium-dependent vasodilation [23]. Altered sympathetic–parasympathetic balance has also been reported in both SLE and ME/CFS.

Immunometabolomic research further links inflammatory pathways to mitochondrial dysfunction, with metabolomic and bioenergetic evidence in ME/CFS [13, 24–26]. Independent studies of cellular bioenergetics corroborate alterations in mitochondrial function and redox balance, while recent multi-tissue transcriptomic and proteomic analyses support widespread mitochondrial dysregulation in ME/CFS [8]. In addition, recent findings suggest that IgG complexes from post-infectious ME/CFS may impair cellular energetics and modulate inflammatory signaling [27], pointing to potential mechanistic parallels between immune-mediated processes and persistent metabolic dysfunction.

#### Hypothesized cross-condition mechanisms requiring validation

This ongoing immunological activity may plausibly contribute to long-term alterations in cellular metabolism, vascular function, and neuroimmune regulation, although this has not yet been established in longitudinal SLE cohorts [28]. Prolonged exposure to inflammatory mediators may impair mitochondrial oxidative phosphorylation and reduce ATP production, thereby compromising cellular energy homeostasis [29]. Mitochondrial dysfunction may be further exacerbated by oxidative and nitrosative stress, resulting in damage to mitochondrial DNA, proteins, and membranes [7, 18, 30].

In SLE, chronic inflammation and oxidative stress may also contribute to endothelial dysfunction, impaired microcirculation and reduce tissue perfusion [9, 31]. In ME/CFS, orthostatic stress has been associated with reduced cerebral blood flow [32, 33], whether comparable perfusion abnormalities contribute to persistent symptoms in SLE requires dedicated investigation. Autonomic dysregulation represents another key component of this proposed framework (in ME/CFS [15]; in SLE [10, 34]). In ME/CFS and related orthostatic-intolerance syndromes, functional autoantibodies targeting adrenergic and muscarinic receptors have been proposed to interfere with autonomic signaling and may contribute to orthostatic intolerance and cardiovascular dysregulation [22, 25, 35]. Evidence for analogous autoantibody-mediated mechanisms in SLE remains limited and inconclusive. Although anti-muscarinic type 3 receptor (M3R) autoantibodies were substantially more prominent in primary Sjögren’s syndrome than in a small SLE comparator group, some SLE samples showed anti-M3R reactivity, particularly in association with anti-SSA/Ro positivity [36]. Their functional relevance for autonomic dysfunction in SLE has not been established. In addition, recent findings suggest that IgG complexes from post-infectious ME/CFS may impair cellular energetics and modulate inflammatory signaling [35], pointing to possible mechanistic analogy with immune complex–mediated dysfunction in SLE [37]. However, whether comparable mechanisms contribute to persistent symptoms in SLE requires direct investigation.

When present simultaneously, these mechanisms may interact in a self-reinforcing manner, linking persistent immune activation with metabolic, vascular, and autonomic dysfunction. Evidence from ME/CFS studies suggests, that in a subset of patients, such interactions could hypothetically culminate in a chronic dysregulation state characterized by hypometabolism [13, 25, 38, 39], neuroinflammation [24, 40], autonomic dysfunction [15], and impaired cerebral perfusion [14, 15, 38], which derives primarily from neuroimaging [14, 15, 24], metabolomic [13], and physiological studies in ME/CFS [14, 25, 38, 40]. Accumulating evidence also highlights skeletal-muscle bioenergetic disturbances as a potential contributor to exertional intolerance in ME/CFS and post-COVID conditions [41], while recent systems-level analyses integrating metabolic, immune, and vascular abnormalities further support the multidimensional nature of this dysregulation [39, 42].

Clinically, the condition described in association with SLE can resemble a range of symptoms seen in ME/CFS, including the ME/CFS-key symptom of post-exertional malaise (PEM) [43], as well as profound fatigue [44], cognitive dysfunction, and orthostatic intolerance. Taken together, these findings support a conceptual model in which metabolic impairment, neuroinflammation, autonomic dysfunction, and vascular dysregulation may interact to sustain chronic symptom burden. This model remains hypothetical in the context of SLE and should be tested in longitudinal studies incorporating immunological, metabolic, autonomic, and vascular measures.

Concepts from network medicine provide one possible theoretical framework for understanding how interacting biological systems might stabilize persistent symptom states. Whether such network dynamics contribute to persistent symptoms in SLE remains entirely speculative and requires empirical investigation.

### Integration with emerging biological frameworks

In the model shown here, proposed conceptual model is consistent with concepts in immunometabolism and neuroimmune interaction and aligns with emerging models linking chronic inflammation, neurological dysfunction, and altered lipid metabolism in post-infectious fatigue syndromes [41, 42, 45]. While supported by metabolomic findings in ME/CFS, this framework remains incompletely validated and requires further empirical investigation.

**Fig. 1:**
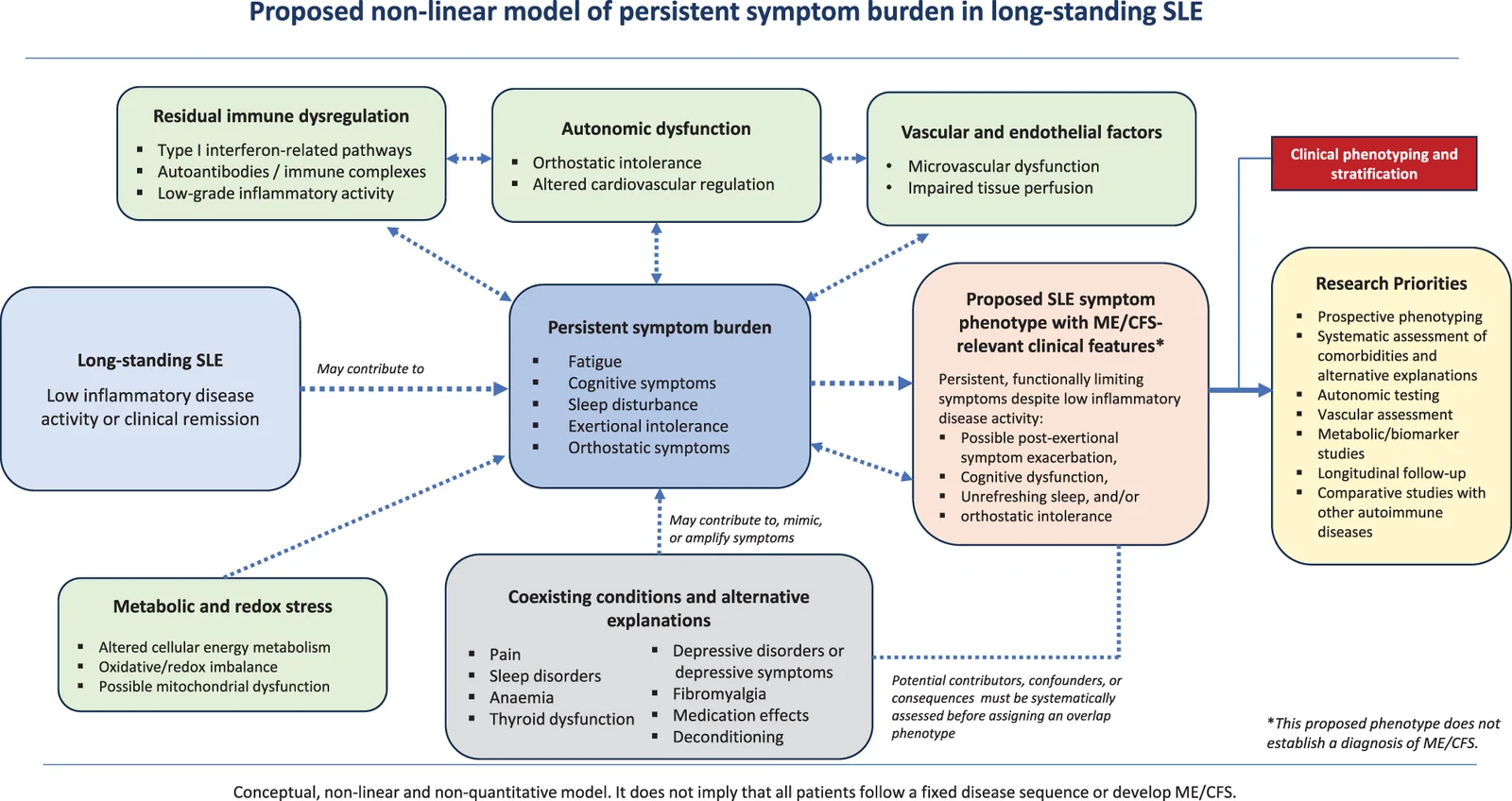
Proposed non-linear, subgroup-focused model of persistent symptom burden in long-standing systemic lupus erythematosus (SLE). In a subset of patients with long-standing SLE, persistent fatigue and functional impairment may occur despite low or fluctuating conventional inflammatory disease activity. Residual immune dysregulation, vascular and endothelial factors, autonomic dysfunction, and metabolic/redox stress may interact bidirectionally and contribute to a proposed SLE–ME/CFS research phenotype. Coexisting or alternative explanations—including pain, sleep disorders, anaemia, endocrine abnormalities, medication effects, depression, deconditioning, and fibromyalgia—must be systematically assessed. The model is conceptual, non-linear, and non-quantitative; it does not imply that all patients follow a fixed disease sequence or develop ME/CFS. Prospective studies are required to define the phenotype, determine its frequency, and identify predictive clinical and biological markers. Solid arrows indicate clinically established associations or disease context; dashed arrows indicate hypothesized or incompletely validated interactions

Figure 1, a schematic diagram, illustrates a proposed pathophysiological conceptual model in which long-standing autoimmune disease, such as systemic lupus erythematosus (SLE), may, in some patients, be associated with progression towards a chronic state of neuroimmunological and metabolic dysregulation resembling myalgic encephalomyelitis/chronic fatigue syndrome (ME/CFS). In the initial phase, autoimmune activation is characterized by autoantibody production, immune complex formation, and systemic inflammation driven by type I interferon signaling and immune cell activation. Over time, persistent inflammatory burden, oxidative stress, and repeated immune activation may contribute to a transition toward a state of chronic immune–metabolic cellular stress. This intermediate phase is marked by mitochondrial dysfunction, endothelial impairment, and autonomic imbalance, potentially mediated by reactive oxygen and nitrogen species, altered nitric oxide signaling, and receptor-targeting autoantibodies. Failure of physiological resolution mechanisms and maladaptive neuroimmune interactions may further promote loss of homeostatic regulation. In the proposed late phase, this process may culminate in a clinical phenotype resembling ME/CFS, characterized by severe fatigue, post-exertional malaise, cognitive dysfunction, orthostatic intolerance, and sleep disturbances. These symptoms are associated with a proposed hypometabolic state, neuroinflammation, dysautonomia, and impaired cerebral perfusion.

### Proposed SLE–ME/CFS-research phenotype for future studies

Adults with established SLE and low inflammatory disease activity/remission who have persistent, functionally limiting fatigue for at least six months, together with post-exertional symptom exacerbation, unrefreshing sleep, cognitive symptoms and/or orthostatic intolerance, after structured assessment of anaemia, thyroid dysfunction, active organ disease, medication effects, major depressive disorder, sleep disorders, fibromyalgia, and deconditioning.

### Clinical implications

This model may help explain the discrepancy between physician-assessed disease activity and patient-reported symptom burden in long-standing SLE [10–12]. Conventional disease activity indices may not adequately capture dysregulatory mechanisms.

If future studies support the approach, it will foster the development of stage-adapted therapeutic strategies that extend beyond immunosuppression and also address mitochondrial dysfunction, autonomic imbalance, and neuroinflammation.

### Testable hypotheses

The proposed model generates several testable hypotheses:

- Long-standing SLE is associated with biomarkers of mitochondrial dysfunction and altered energy metabolism
- Autonomic dysfunction and impaired cerebral perfusion are prevalent in SLE patients with severe fatigue
- A subset of SLE patients fulfills diagnostic criteria for ME/CFS or exhibits overlapping biomarker profiles
- Longitudinal progression from inflammatory to dysregulatory phenotypes can be demonstrated

An additional testable hypothesis is that specific clinical and biological susceptibility factors distinguish patients who develop persistent symptom burden despite low inflammatory disease activity from those who do not. Candidate susceptibility factors include cumulative inflammatory burden, vascular vulnerability, autonomic phenotype, genetic and epigenetic background, and environmental or psychosocial modifiers. Identifying such factors may help explain interindividual heterogeneity, facilitate early risk stratification, and provide a basis for prospective longitudinal studies aimed at defining biologically meaningful patient subgroups.

Potential measurement dimensions:

- SLE/inflammatory status: Systemic Lupus Erythematosus Disease Activity Index (SLEDAI)/ British Isles Lupus Assessment Group (BILAG), complement, anti-dsDNA, interferon signature, organ activity
- Fatigue and exercise intolerance: FACIT-Fatigue, PROMIS Fatigue, functional exercise parameters; do not equate Post-Exertional Malaise (PEM) solely with “fatigue”
- Autonomic function: active standing test or tilt table test, heart rate and blood pressure responses
- Cognition/sleep: standardized brief neuropsychological tests, validated sleep instruments
- Vascular/metabolic markers: present only as exploratory candidates, not as established markers

## Limitations

This Commentary presents a conceptual framework rather than a data-driven model and should therefore be interpreted as a hypothesis-generating synthesis. The proposed subgroup and the biological interactions described here have not been validated in longitudinal clinical cohorts and do not imply a causal sequence or fixed disease trajectory. The model is intended to provide a testable framework for future translational research rather than a definitive explanation of persistent symptom burden in SLE.

Several of the proposed mechanisms, including alterations in cellular energetics, cerebral perfusion, and autonomic regulation, are supported more strongly by evidence from ME/CFS than by direct evidence from SLE. Throughout this Commentary, such cross-condition inferences have therefore been explicitly distinguished from established SLE findings. Whether comparable mechanisms contribute to persistent symptoms in SLE remains to be determined in prospective studies incorporating immunological, autonomic, vascular, and metabolic phenotyping. SLE is a highly heterogeneous disease, and the proposed framework is not intended to apply to all patients. Instead, it is restricted to a hypothesised subgroup with persistent functional impairment despite low inflammatory disease activity or clinical remission. At present, the prevalence, clinical boundaries, and biological characteristics of this subgroup remain unknown.

Finally, symptom overlap with ME/CFS should not be interpreted as evidence that these conditions are identical. Persistent fatigue, cognitive symptoms, sleep disturbance, and orthostatic intolerance may also arise from active disease, accumulated organ damage, anaemia, endocrine disorders, medication effects, depression, fibromyalgia, pain syndromes, sleep disorders, or deconditioning. Careful clinical assessment of alternative explanations remains essential before considering the proposed research phenotype.

## Conclusions

Persistent symptom burden despite low inflammatory disease activity remains an important unresolved clinical problem in systemic lupus erythematosus (SLE). The framework proposed in this Commentary suggests that, in a biologically distinct subgroup of patients with long-standing SLE, persistent functional impairment may reflect interacting immune, autonomic, vascular, metabolic, and neurocognitive mechanisms that are only partially captured by conventional measures of inflammatory disease activity.

Rather than proposing a fixed disease continuum or a new diagnostic entity, this model is intended as a subgroup-focused, non-linear, and hypothesis-generating framework. It integrates established evidence from SLE with carefully distinguished mechanistic inference from ME/CFS research in order to identify biologically plausible pathways that warrant direct investigation in SLE.

The proposed phenotype should not be defined by fatigue alone but by persistent functional limitation together with characteristic symptom patterns such as post-exertional symptom exacerbation, cognitive dysfunction, unrefreshing sleep, or orthostatic intolerance after structured assessment of alternative explanations and relevant comorbidities.

Future research should prioritize prospective longitudinal studies combining detailed clinical phenotyping with autonomic, vascular, metabolic, and immunological assessment. Particular emphasis should be placed on identifying susceptibility factors, defining biologically meaningful patient subgroups, and distinguishing mechanisms related to persistent inflammation from those reflecting broader systemic dysregulation. Such studies may ultimately improve patient stratification, biomarker development, and supportive management for patients whose symptom burden remains disproportionate to conventional measures of disease activity.

## Data Availability

Not applicable.
